# Effect of ATM and HDAC Inhibition on Etoposide-Induced DNA Damage in Porcine Early Preimplantation Embryos

**DOI:** 10.1371/journal.pone.0142561

**Published:** 2015-11-10

**Authors:** HaiYang Wang, YiBo Luo, ZiLi Lin, In-Won Lee, Jeongwoo Kwon, Xiang-Shun Cui, Nam-Hyung Kim

**Affiliations:** Department of Animal Sciences, Chungbuk National University, Naesudong-ro, Seowon-gu, Cheongju-si, 362–763, Chungcheongbuk-do, Korea; Institute of Zoology, Chinese Academy of Sciences, CHINA

## Abstract

Oocyte maturation and embryonic development are sensitive to DNA damage. Compared with somatic cells or oocytes, little is known about the response to DNA damage in early preimplantation embryos. In this study, we examined DNA damage checkpoints and DNA repair mechanisms in parthenogenetic preimplantation porcine embryos. We found that most of the etoposide-treated embryos showed delay in cleavage and ceased development before the blastocyst stage. In DNA-damaged embryos, the earliest positive TUNEL signals were detected on Day 5 of *in vitro* culture. Caffeine, which is an ATM (ataxia telangiectasia mutated) and ATR (ataxia telangiectasia and Rad3-related protein) kinase inhibitor, and KU55933, which is an ATM kinase inhibitor, were equally effective in rescuing the etoposide-induced cell-cycle blocks. This indicates that ATM plays a central role in the regulation of the checkpoint mechanisms. Treating the embryos with histone deacetylase inhibitors (HDACi) increased embryonic development and reduced etoposide-induced double-strand breaks (DSBs). The mRNA expression of genes involved in non-homologous end-joining (NHEJ) or homologous recombination (HR) pathways for DSB repair was reduced upon HDACi treatment in 5-day-old embryos. Furthermore, HDACi treatment increased the expression levels of pluripotency-related genes (*OCT4*, *SOX2* and *NANOG*) and decreased the expression levels of apoptosis-related genes (*CASP3* and *BAX*). These results indicate that early embryonic cleavage and development are disturbed by etoposide-induced DNA damage. ATMi (caffeine or KU55933) treatment bypasses the checkpoint while HDACi treatment improves the efficiency of DSB repair to increase the cleavage and blastocyst rate in porcine early preimplantation embryos.

## Introduction

DNA double-strand breaks (DSBs) can be induced by endogenous metabolites or metabolic intermediates [1] as well as by exogenous factors such as UV [2], ionizing radiation (IR; including X-rays and γ-rays), chemical drugs (such as doxorubicin, bleomycin, MLN4924, and etoposide), and physical inducers (such as laser micro-beam dissection) [3–6]. The occurrence of DSBs activates DNA damage checkpoints and DNA repair mechanisms. DNA damage checkpoint mechanisms arrest cell division until all DSBs are repaired. However, if abnormal DNA DSBs, such as the programmed DSBs, are not repaired immediately they can induce chromatin remodeling, cell cycle arrest, cell cycle delay, apoptosis, or other forms of cell death [7]. DSBs are known to affect oocyte maturation in multiple species [4–6,8]. Compared with oocytes, little is known about the response to DSBs in early preimplantation embryos.

In mammals, two molecular pathways are involved in DSBs repair: homologous recombination (HR) and non-homologous end-joining (NHEJ) [9,10]. In response to DNA damage, the protein kinases ATM (ataxia telangiectasia mutated) and ATR (ataxia telangiectasia and Rad3-related protein) phosphorylate checkpoint kinase 2 (CHK2). The activated CHK2 pathway then arrests the cell cycle [11]. ATM and ATR can also phosphorylate the histone H2AX (γH2AX) at the sites of DSB formation [12,13]. This phosphorylation involves large chromatin domains forming nuclear foci that are easily detected by immunostaining [14]. The phosphorylation of histone H2AX is critical for DSB repair because H2AX anchors some important initiator proteins that are required for both HR (e.g., *RAD51*, *MRE11A*, *BRCA1*) and NHEJ (e.g., *53BP1*, *PRKDC*, *XRCC6*) pathways, which can be colocalized with γH2AX at the sites of DSBs [15,16]. After DSBs are repaired, the checkpoint proteins become inactivated to allow cell cycle progression to resume.

Caffeine, a methylxanthine, has been widely used to study ATM and ATR signaling because it inhibits these kinases *in vitro* and allows DNA-damaged cells to bypass checkpoints *in vivo* [2,17–19]. Thus, caffeine is thought to overcome the checkpoint by preventing phosphorylation of ATM and ATR substrates. KU55933 is a potent inhibitor of ATM with a half maximal inhibitory concentration (IC_50_) of 13 nmol/L, and is highly specific to ATM compared with other PIKKs (phosphoinositide-3-kinase-related protein kinases) [20]. It prevents the activation of selective ATM targets following DNA DSBs, and prevents the phosphorylation of ATM-dependent DNA damage checkpoints in response to ionizing radiation [20]. The relative roles of ATM (caffeine- and KU55933-sensitive) and ATR (caffeine-sensitive and KU55933-insensitive) in response to etoposide-induced DSBs in porcine embryos can be determined when KU55933 is used in conjunction with caffeine [2].

Scriptaid is a novel HDACi with low toxicity and enhanced transcriptional activity [21]. Scriptaid treatment significantly improves the development of cloned embryos and alleviates aberrant gene expression, as demonstrated in mice and cattle [22,23]. In somatic cells, histone modifications play important roles in DSBs repair. For instance, the acetylation of histone site H4K16 is critical for the DNA damage response and DSB repair by the HR and NHEJ pathways [24]. In addition, acetylation of histone sites H4K5 and H4K12 at the sites of DSBs facilitates the recruitment of the RAD51 protein [25]. It has also been shown that Gcn5, which is a histone acetyltransferase (HAT), can interact with γH2AX at the site of DSBs and then acetylate various lysine residues on histone H3 (including H3K9, H3K14, H3K18, and H3K23) [26]. H3K9Ac and H3K56Ac are reduced in response to DNA damage in human cells [27]. Based on these discoveries, we hypothesized that increasing histone acetylation by HDACi treatment could facilitate DNA damage repair in porcine early preimplantation embryos.

Etoposide is a topoisomerase II (TOP2) inhibitor that causes DSB DNA damage in somatic cells as well as in oocytes, even at low concentrations (5 μg/mL) [4,28]. Exposure to etoposide has been used to study DNA damage signaling and repair mechanisms in both somatic cells and oocytes [4]. However, to our knowledge, there has been no research on the effect of etoposide in porcine embryos. Therefore, the objectives of this study were: (1) to evaluate the effect of DNA damage on the development of early embryos; and (2) to assess the effect of ATMi (caffeine or KU55933) and HDACi (scriptaid) treatment on the DNA damage response and development in preimplantation embryos.

## Materials and Methods

### Oocyte Collection and Culture

All chemicals used in this study were purchased from Sigma-Aldrich, unless otherwise stated. All animal studies were performed after receiving the approval of the Institutional Animal Care and Use Committee (IACUC) of Chungbuk National University, Korea. Porcine ovaries were provided by the local slaughterhouse (Farm story dodarm B&F, Umsung, Chungbuk, Korea) and were transported to our laboratory at 25°C in Dulbecco’s phosphate-buffered saline supplemented with 75 μg/L penicillin G and 50 μg/L streptomycin sulfate. Cumulus–oocyte complexes (COCs) were aspirated from follicles (diameter approximately 2–8 mm) and were washed three times with HEPES-buffered Tyrode’s medium containing 0.1% (w/v) polyvinyl alcohol (PVA)(HEPES is 4-(2-hydroxyethyl)-1-piperazineethanesulfonic acid). The collected COCs were matured in tissue culture medium 199 (Gibco) supplemented with 0.1 g/L sodium pyruvate, 0.6 mM L-cysteine, 10 ng/mL epidermal growth factor, 10% porcine follicular fluid (v/v), 10 IU/mL luteinizing hormone, and 10 IU/mL follicle-stimulating hormone for 44 h at 38.5°C in 5% CO_2_ and humidified air. After maturation, the cumulus cells were removed by pipetting in the presence of 0.1% hyaluronidase (w/v) for 2–3 min.

### Oocyte Activation and Embryo Culture

The oocytes were activated parthenogenetically by a 5-min treatment with 50 μM Ca^2+^ ionophore (A23187, Sigma-Aldrich). After 3 h of culture in porcine zygote medium 5 (PZM-5) supplemented with 7.5 μg/mL cytochalasin B (Sigma), the embryos were washed several times in PZM-5 supplemented with 0.4% (w/v) bovine serum albumin (BSA) and cultured in a humidified atmosphere of 5% CO_2_ and 95% air at 38.5°C.

### Drug Treatments

KU55933 (Selleckchem) was used as an ATM inhibitor, caffeine (Sigma) as an ATR and ATM inhibitor, and scriptaid (Sigma) as an HDAC inhibitor. To investigate DNA damage, etoposide (100 mg/mL stock in dimethyl sulfoxide; stored at 4°C until required) was added to the IVM or IVC medium to a final concentration of 0 (controls), 25, 50, and 100 μg/mL. To induce DSBs in oocytes, GV oocytes were cultured in IVM medium containing 25 μg/mL of etoposide for 5 h, after which the cumulus cells were removed to determine the levels of γH2AX or ATM-p. To induce DSBs in embryos, parthenogenic embryos were cultured in IVC medium containing 0 (controls), 25, 50, or 100 μg/mL of etoposide for 5 h starting after removal from cytochalasin B. Parthenogenic embryos were treated or not (control) with KU55933 (10 μM), caffeine (5 mM), or scriptaid (500 μM) for 20 h starting after Ca^2+^ ionophore treatment.

### Immunofluorescence Analysis

Oocytes or embryos were washed with phosphate-buffered saline (PBS), fixed in 3.7% paraformaldehyde (w/v) prepared in PBS containing 0.1% PVA, and permeabilized with 1% Triton X-100 (v/v) for 1 h at 37°C. The samples were blocked with 1% BSA (w/v) for 1 h, incubated overnight with different antibodies at 4°C in a blocking solution, and washed with 1% BSA. The oocytes or embryos were then incubated overnight with anti-γH2AX (pS139, 1:100; Cell Signaling Technology), anti-ATM (pS1981, 1:100; Cell Signaling Technology), and anti-Histone H3 (acetyl K9, Abcam, Cambridge) antibodies at 4°C overnight. The oocytes or embryos were washed three times with PBS containing 1% BSA and were labeled with FITC/TR-conjugated antibody (1:100) for 1 h at room temperature (FITC/TR is fluorescein isothiocyanate and Texas Red). The oocytes were then counterstained with 5 μg/mL Hoechst 33342 for 15 min, washed three times with PVA–PBS, mounted on a glass slide, and examined using an LSM 710 META confocal laser-scanning microscope (Zeiss, Jena, Germany).

### Fluorescence Intensity Analysis

For fluorescence intensity analysis, samples of control oocytes/embryos and treated oocytes/embryos were mounted on the same glass slide. ImageJ software (v1.47) was used to define a region of interest (ROI), and the average fluorescence intensity per unit area within the ROI was determined. Independent measurements using identically sized ROIs were made for the cell nucleus. The average values of all measurements were used to compare the final average intensities between control and treated oocytes or embryos.

### Reverse Transcription Quantitative Polymerase Chain Reaction (RT-qPCR) with SYBR Green

Total RNA was extracted from groups of 30 (Day 5) and 20 (Day 7) porcine embryos using the Dynabeads mRNA Direct Kit (Dynal Asa, Oslo, Norway) according to the manufacturer’s instructions. First-strand complementary DNA (cDNA) was synthesized by reverse transcription of mRNA using the Oligo (dT) 12–18 primer and SuperScript TM III Reverse Transcriptase (Invitrogen Co., Grand Island, NY). Real-time PCR (also called quantitative PCR) was performed using a CFX96 Touch Real-time PCR Detection System (Bio-Rad) in a final reaction volume of 20 μL including SYBR Green, a fluorophore that binds to double-stranded DNA (qPCR kit from Finnzymes, Finland). The PCR conditions were as follows: 95°C for 10 min followed by 39 cycles of 95°C for 30 s, 60°C for 30 s, and 72°C for 25 s, and a final extension at 72°C for 5 min. Finally, gene expression was quantified using the 2^-ΔΔCt^ method, and normalized against the mRNA levels of glyceraldehyde 3-phosphate dehydrogenase (GAPDH). The primers used to amplify each gene are shown in Table 1.

**Table 1:** Primers used for real-time reverse transcription-PCR.

Gene	Primer sequences (5'-3')	Accession number
*BCLXL*	F: TTTTCTCCTTCGGTGGGG	NM_214285.1
	R: GCATTGTTTCCGTAGAGTTCC	
*BAX*	F: T ACCTT ACCCTGGGAGTGGC	XM_001928147.2
	R: GGAAAACCTCCTCCGTGTC	
*CASP3*	F: GAAGACCATAGCAAAAGGAGCA	NM_214131.1
	R: TTTGGGTTTGCCAGTTAGAGTT	
*NANOG*	F: TTCCTTCCTCCATGGATCTG R:	NM_001129971.1
	ATCTGCTGGAGGCTGAGGTA	
*SOX2*	F: CGCAGACCTACATGAACG	NM_001123197.1
	R: TCGGACTTGACCACTGAG	
OCT4	F: AAGCAGTGACTATTCGCAAC	NM_001113060.1
	R: CAGGGTGGTGAAGTGAGG	
*ATM*	F: CCGGTGTTTTGGGAGAGTGT	NM_001123080.1
	R: CTTCCGACCAAACTCAGCGT	
*ATR*	F: TGAGCTCCAGTGTTGGCATC	XM_003132459.3
	R: GCCAGTTCTCAGTGTGGTCA	
*RAD51*	F: CTTCGGTGGAAGAGGAGAGC	NM_001123181.1
	R: CGGTGTGGAATCCAGCTTCT	
*BRCA1*	F: TGCTAAATCCGGAACAAAACACA	XM_003358030.1
	R: CTGGTGGAACGATCCAGAGAT	
*MRE11A*	F: GGAGGATGTTGTCCTGGCTG	XM_003129789.2
	R: AGACGTTCCCGTTCTGCATT	
*XRCC6*	F: ACGGAAGGTGCCCTTTACTG	NM_001190185.1
	R: TGCAGCACTGGGTTCTCAAA	
*PRKDC*	F: ATTCTTTGTCGGGAGCAGCA	XM_001925309.4
	R: CCTAGCTGTGTGGCACATGA	
*TP53BP1*	F: GGGAAAGGGGGAGTTCGTG	XM_001925938.4
	R: CTCACGCTCGTGCTAGAGAT	
*GAPDH*	F: GCCA TCACCA TCTTCCAGG R:	NM_001206359.1
	TCACGCCCATCACAAACAT	

F, forward; R, reverse.

### Terminal Deoxynucleotidyl Transferase (TdT)-Mediated dUTP-Digoxigenin Nick End Labeling (TUNEL) Assay

Embryos were washed three times with PBS (pH 7.4) containing 1 mg/mL polyvinyl pyrrolidone (PBS/PVP). After fixation with 3.7% paraformaldehyde prepared in PBS for 1 h at room temperature (RT), the embryos were washed with PBS/PVP and permeabilized by incubation in 0.5% Triton X-100 for 1 h at RT. The embryos were then washed twice with PBS/PVP and incubated with fluorescein-conjugated dUTP and terminal deoxynucleotidyl transferase (In Situ Cell Death Detection Kit, Roche, Mannheim, Germany) in the dark for 1 h at 37°C. Blastocysts were counterstained with Hoechst 33342 (bis-benzimide H33342 trihydrochloride, Sigma Life Science) to label all nuclei, washed with PBS/PVP, mounted with slight coverslip compression, and examined under an Olympus fluorescence microscope.

### Statistical Analysis

Each experiment was performed at least three times and at least 30 oocytes or 20 embryos were examined each time. Statistical analyses were performed with the SPSS software package (Version 11.5; SPSS). Analysis of variance (ANOVA) was adopted for statistical analyses. Differences between treated groups were evaluated with the Duncan multiple comparison test. Data are expressed as mean ± SD, and a p-value < 0.05 was considered statistically significant.

## Results

### Early Embryonic Cleavage and Development Is Disturbed by Etoposide-Induced DNA DSBs

**Fig 1:**
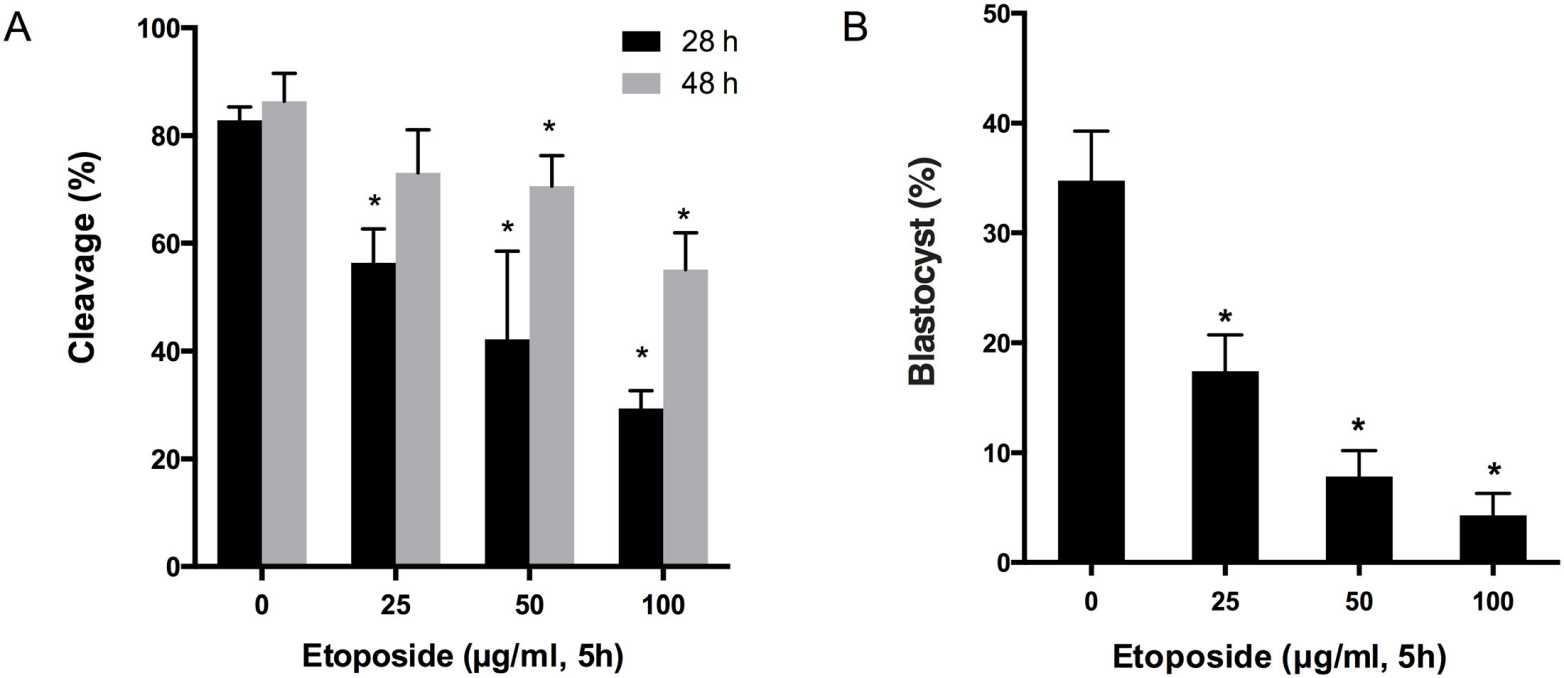
Development of embryos treated with various concentrations of etoposide. Cleavage (**A**) and blastocyst (**B**) rates of porcine embryos that were unexposed (control, 0 μg/mL etoposide) or exposed to 25, 50, or 100 μg/mL etoposide for 5 h. Cleavage rates were determined at 28 h and 48 h after parthenogenetic activation. Blastocyst rates were detected at day 7 of embryonic culture. Data are from 3 independent replicates with a minimum of 30 embryos in each group. Asterisks denote a significant difference from the controls (* p < 0.05).

In the non-exposed control group (0 μg/mL etoposide), no embryos had cleaved at 18 h after parthenogenetic activation, whereas 82.87% and 86.38% had cleaved at 28 h and 48 h, respectively (Fig 1A). However, only 56.41%, 42.20%, and 29.35%, respectively, of the 25, 50, or 100 μg/mL etoposide-exposed embryos had cleaved at 28 h. Forty-eight hours after treatment with 25, 50, or 100 μg/mL etoposide, the rate of cleavage increased to 73.09%, 70.62%, and 64%, respectively (Fig 1A). Although a significant proportion of etoposide-treated embryos could develop beyond the first cleavage stage, development to the blastocyst stage at Day 7 was dramatically reduced (Fig 1B). Indeed, development to blastocysts was almost completely blocked by 50 (7.83%) and 100 μg/mL (4.29%) of etoposide exposure, while it was approximately 50% reduced by 25 μg/mL treatment (17.41%), compared with the control embryos (34.77%) (Fig 1B).

### Etoposide Treatment Affects Embryo Quality and Increases Apoptosis

**Fig 2:**
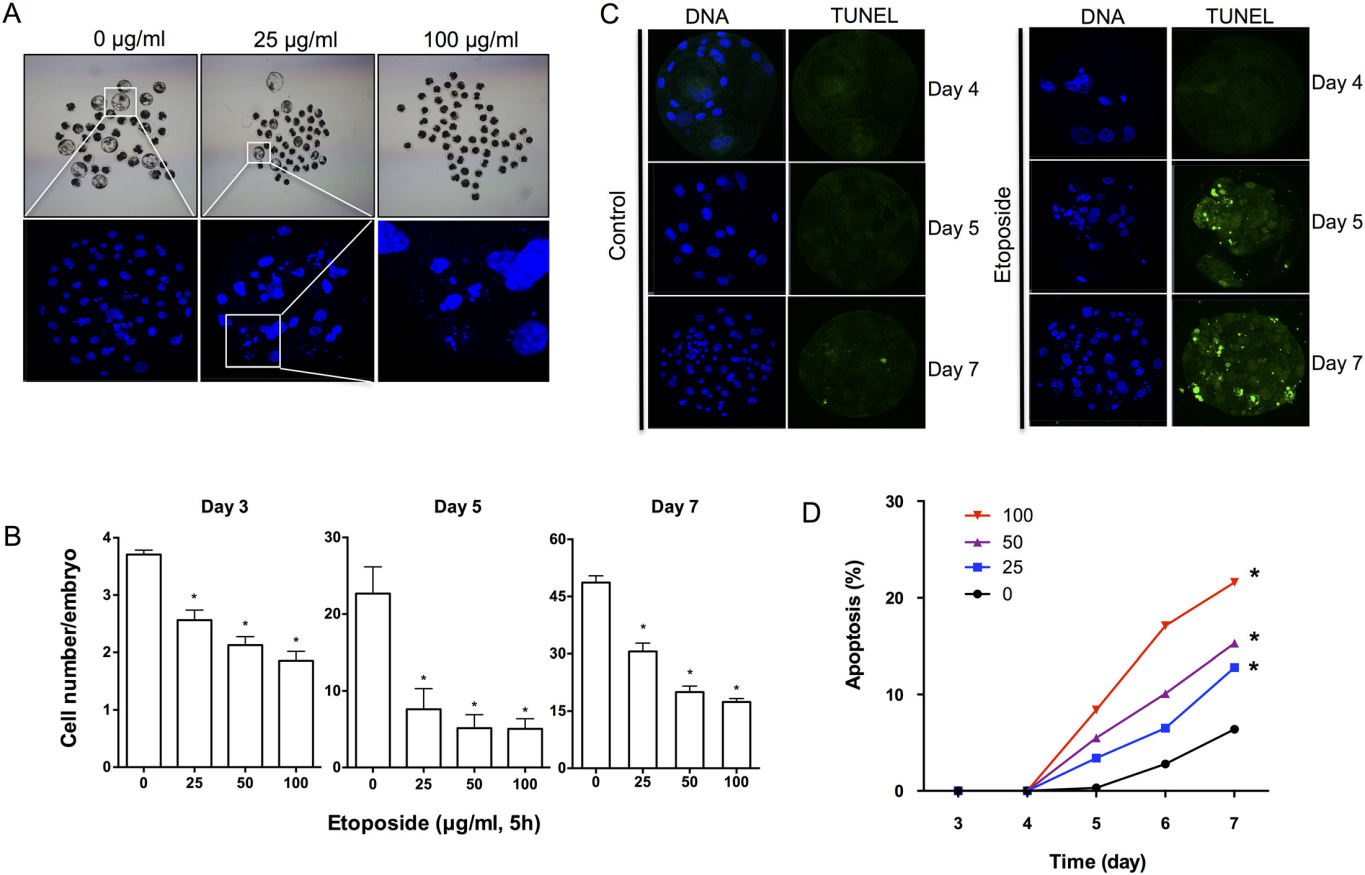
Etoposide treatment resulted in poor-quality embryos. (**A**) Schematic diagram of 7-day-old embryos. The blastocyst rates and cell numbers were lower after 25 and 100 μg/mL etoposide treatment. We found that the etoposide-treated embryos contained multiple micronuclei. **(B)** Cell numbers in etoposide-treated (0, 25, 50, 100 μg/mL) embryos fixed on day 3, 5, and 7 of culture. Data are from three independent replicates and at least 20 embryos were analyzed in each experiment. **(C)** TUNEL-positive cells were detected in etoposide-treated (25 μg/mL) embryos at day 5 of culture and TUNEL staining further increased by day 7. (**D**) Apoptotic rate (number of apoptotic cells/number of total cells) in embryos from day 3 to day 7. Embryos were not treated (control) or treated with 25 μg/mL etoposide. Asterisks denote a significant difference from unexposed controls (* p < 0.05). Values are means ± SD of 3 independent experiments.

The blastocyst cell number is an important parameter in assessing the blastocyst quality. We evaluated the total number of cells by Hoechst 33342 staining. The results indicated that the total cell number of blastocysts was significantly higher among the etoposide-treated embryos (25 μg/mL) than among the non-treated embryos (Fig 2A). Additionally, we observed that the etoposide-treated blastocysts appeared to be morphologically abnormal and had more micronuclei compared with those of the control group (Fig 2A). Most of the etoposide-treated (25, 50, or 100 μg/mL) embryos were arrested at the 4–8-cell stage, although most of the embryos in the control group had already reached the morula stage after 5 days of development (Fig 2B). Furthermore, mRNA expression of three pluripotency-related genes, *OCT4*, *SOX2* and *NANOG*, was lower in etoposide-treated (25 μg/mL) embryos than in non-treated embryos at the blastocyst stage. These findings indicate that the embryo quality was influenced by etoposide treatment.

The rate of apoptosis was calculated by dividing the number of cells with TUNEL-positive nuclei by the total embryo cell number. We analyzed five time-points (Day 3, 4, 5, 6, and 7) to determine the time-point when apoptosis was initially increased. The results indicated that TUNEL-positive nuclei appeared on Day 5 after etoposide treatment, and the staining was further enhanced by Day 7 (Fig 2C and 2D). However, no TUNEL staining was detected prior to Day 5. The percentage of TUNEL-positive nuclei appeared to remain constant from Day 5 to Day 7 in the control group, but it increased to a significantly higher level (p < 0.05) between Day 5 and Day 7 in the etoposide-treated groups (Fig 2C and 2D).

### Histone H2AX Is Activated in Oocytes and Early Preimplantation Embryos with DNA DSBs

**Fig 3:**
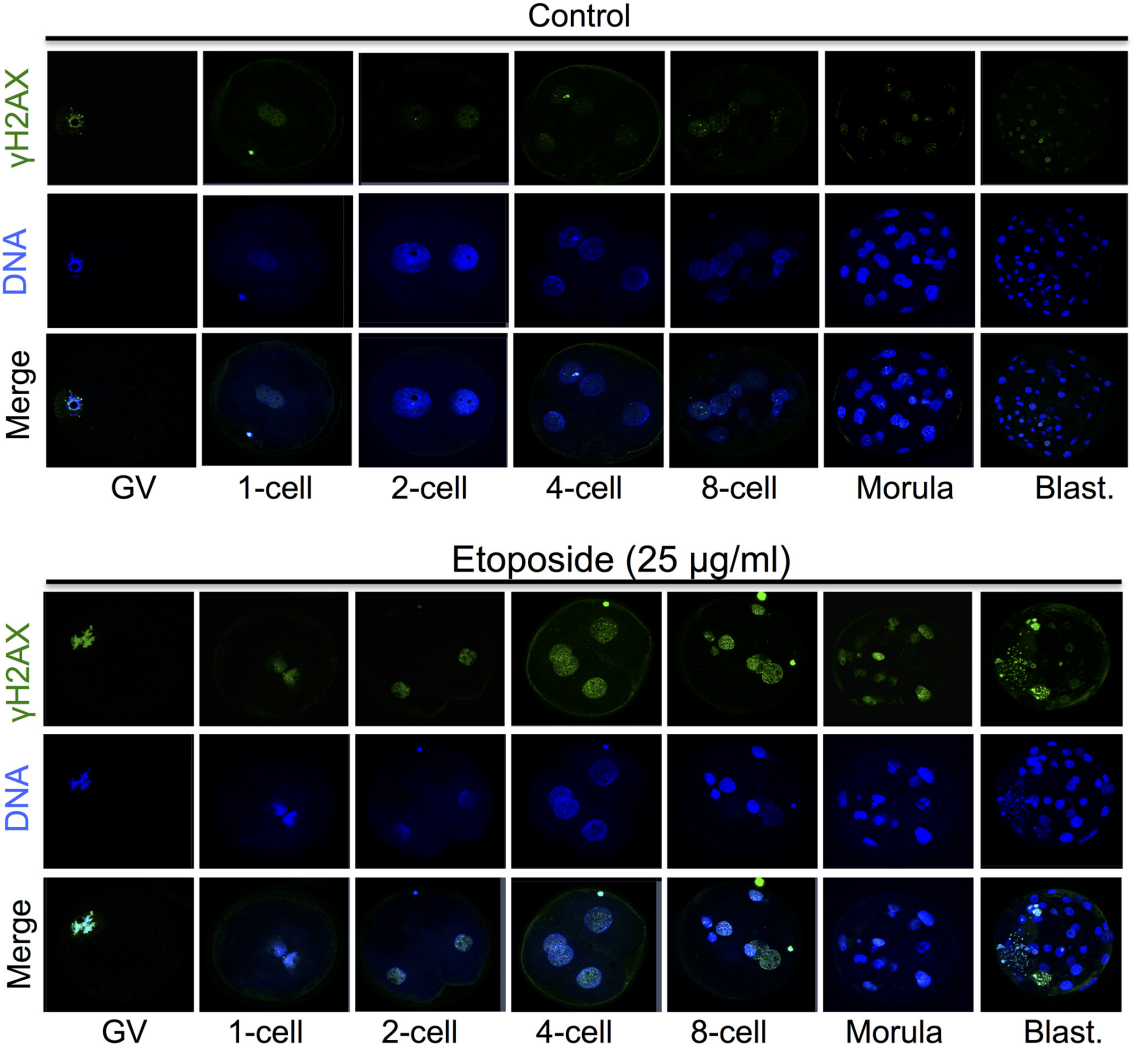
Treatment with 25 μg/mL etoposide for 5 h causes the activation of histone H2AX (γH2AX) at the sites of DNA damage. Representative fluorescent images of oocytes and embryos that were unexposed (control) or exposed to etoposide (25 μg/mL) for 5 h. Cells were immunostained with an antibody against γH2AX. Hoechst was used to stain DNA.

To determine whether the DNA repair mechanisms are activated in porcine preimplantation embryos, we examined γH2AX, which forms a platform for the recruitment of the necessary DNA damage checkpoint and repair factors (Fig 3). The presence of phosphorylated H2AX at Ser139 (γH2AX) was examined after treatment with 25 μg/mL etoposide. We observed that levels of γH2AX were low in the non-exposed control group, but increased in oocytes and preimplantation embryos treated with 25 μg/mL etoposide (Fig 3). Our results indicate that the DNA repair mechanism is functional in porcine preimplantation embryos.

### Caffeine and KU55933 Treatment Increases Development and Reduces Phosphorylation of H2AX in Embryos

**Fig 4:**
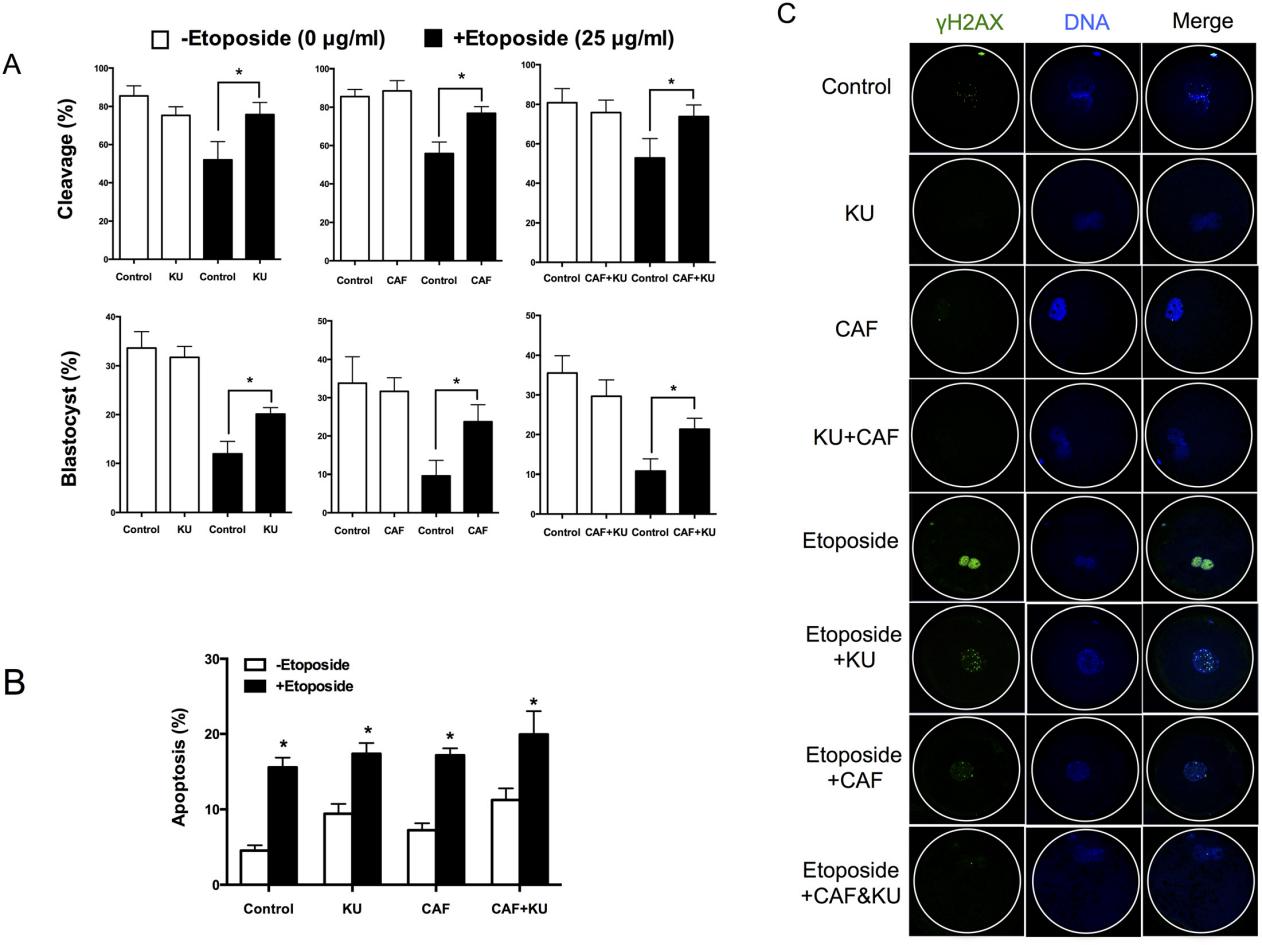
The effect of caffeine and KU55933 on embryonic development after etoposide treatment. Embryos were treated with etoposide (25 μg/mL) with or without caffeine (5 mM) and/or KU55933 (10 μM). (A) Cleavage and blastocyst rates were detected after 28 h and 7 days of culture, respectively. Data are from 3 independent replicates with a minimum of 20 embryos in each group. * P < 0.05. (B) Apoptosis rates were detected at day 7 of culture. Asterisks denote a significant difference from the controls, which were not treated with etoposide (* p < 0.05). (C) The levels of γH2AX in the nuclei of 1-cell embryos.

The effect of caffeine and KU55933 treatment was evaluated in control and etoposide-exposed early preimplantation embryos. Cleavage rates (28 h after parthenogenetic activation) were significantly increased (p < 0.05) in caffeine- and KU55933-treated embryos after induction of DNA damage by etoposide treatment (25 μg/mL, 5 h) (Fig 4A). Interestingly, compared with non-treated embryos, caffeine and KU55933 treatment resulted in a 1.68-fold (11.97% *vs*. 20.11%) and 2.46-fold (9.63% *vs*. 23.71%) increase, respectively, in development to the blastocyst stage of etoposide-exposed (25 μg/mL) embryos (Fig 4A). However, caffeine or KU55933 treatment did not reduce the etoposide treatment-induced apoptosis at the blastocyst stage (Fig 4B). The capacity of KU55933 to ameliorate checkpoint recovery after etoposide treatment suggests that ATM is implicated in this response. The accumulation of γH2AX in the nuclei of one-cell embryos in response to etoposide was blocked to an equal extent by KU55933 and caffeine (Fig 4C).

### Etoposide Treatment Activates an ATM-Dependent DNA Damage Checkpoint

**Fig 5:**
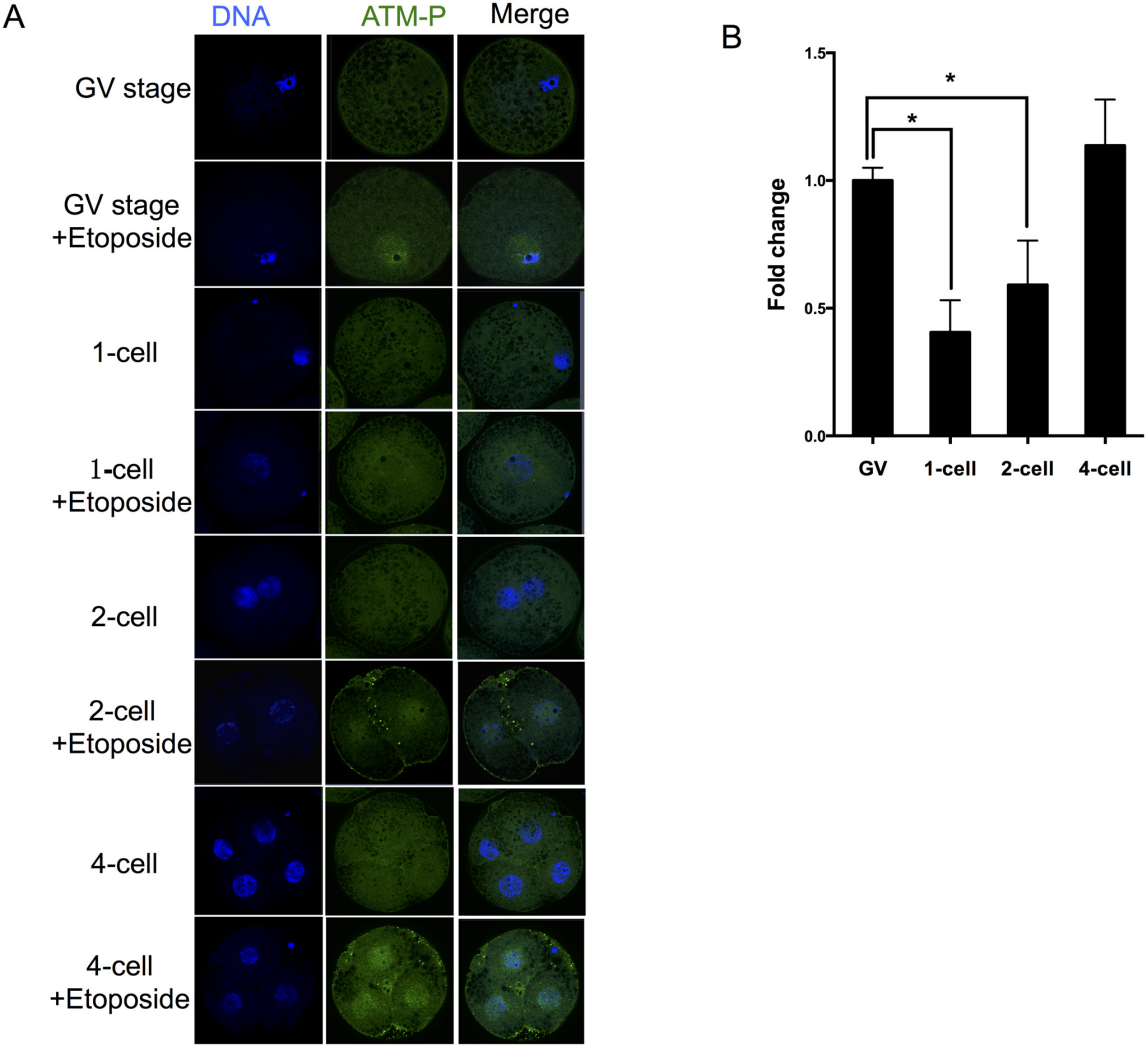
Phosphorylated ATM in the oocytes and preimplantation-stage embryos. (A) Immunolabeling for active ATM at 100 μg/mL etoposide treatment. (B) Fold change in ATM-p was determined using the formula F-F_0_/F_GV_-F_0_, where F is the mean value of each etoposide treatment, and F_0_ is the control value where no etoposide is used (0 μg/mL). F_GV_ is the value of GV stage exposure to 100 μg/mL etoposide. This formula was used to be able to disregard nonspecific staining, which allows for a better comparison of the different cell types.

Having found that ATM is likely to be involved in the response to etoposide-induced DNA damage, we next sought to define the levels of ATM activation (phosphorylated at serine 1981, ATM-p) upon etoposide exposure. In the non-treated control group, ATM-p was not detected in porcine GV-stage oocytes and embryos (Fig 5A). After exposure to 100 μg/mL etoposide, phosphorylated ATM was detected. In one- and two-cell embryos, only a limited amount of phosphorylated ATM was detected after etoposide-exposure, and the levels were clearly lower when compared with those of fully grown oocytes or late four-cell embryos (Fig 5B).

### HDACi Treatment Stimulates Development and Reduces Phosphorylated H2AX in Porcine Embryos

**Fig 6:**
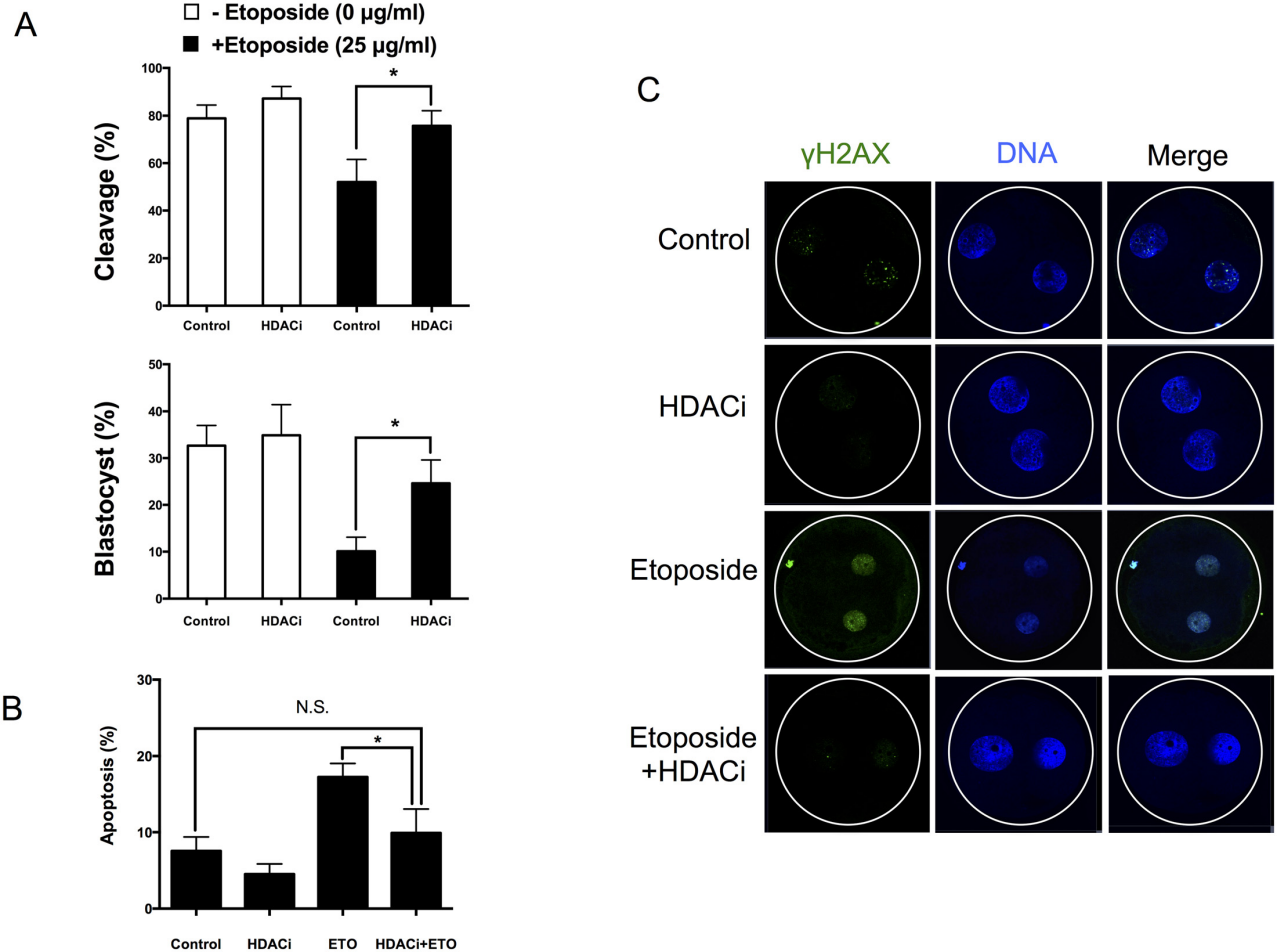
HDACi treatment increased development and reduced phosphorylated H2AX in cleaved embryos. Non-treated (-Etopodide; white bars) or etoposide-treated (+Etopodide; black bars) embryos were subjected to HDACi or vehicle (control). The levels of cleavage (A) are determined 28 h after activation. * P < 0.05. The levels of blastocyst formation (B) are determined at day 7 of embryonic culture * P < 0.05; N.S., not statistically significant (P > 0.05). (C) The levels of γH2AX in the nuclei of cleaved embryos. The concentration of etoposide used in this experiment was 25 μg/mL. Data are from 3 independent replicates with 30 embryos in each group.

Since we observed that the DNA damage checkpoint mechanisms in embryos do not appear to be functioning efficiently, we next sought to define whether HDACi treatment enhances DSB repair. The effect of HDACi treatment was evaluated in non-exposed (control) and etoposide-exposed embryos. Cleavage rates were significantly increased in HDACi-treated embryos subjected to DNA-damaging conditions (25 μg/mL etoposide treatment) (Fig 6A). Interestingly, HDACi treatment resulted in a 2.44-fold (10.11% *vs*. 24.63%) increase in development to the blastocyst stage of etoposide-treated embryos, compared with non-treated embryos (Fig 6A). In addition, HDACi treatment decreased the occurrence of DSB-induced apoptosis at the blastocyst stage (Fig 6B). HDACi treatment also reduced the levels of γH2AX in cleaved etoposide-exposed embryos (Fig 6C).

### HDACi Treatment Leads to Increased Histone Acetylation

**Fig 7:**
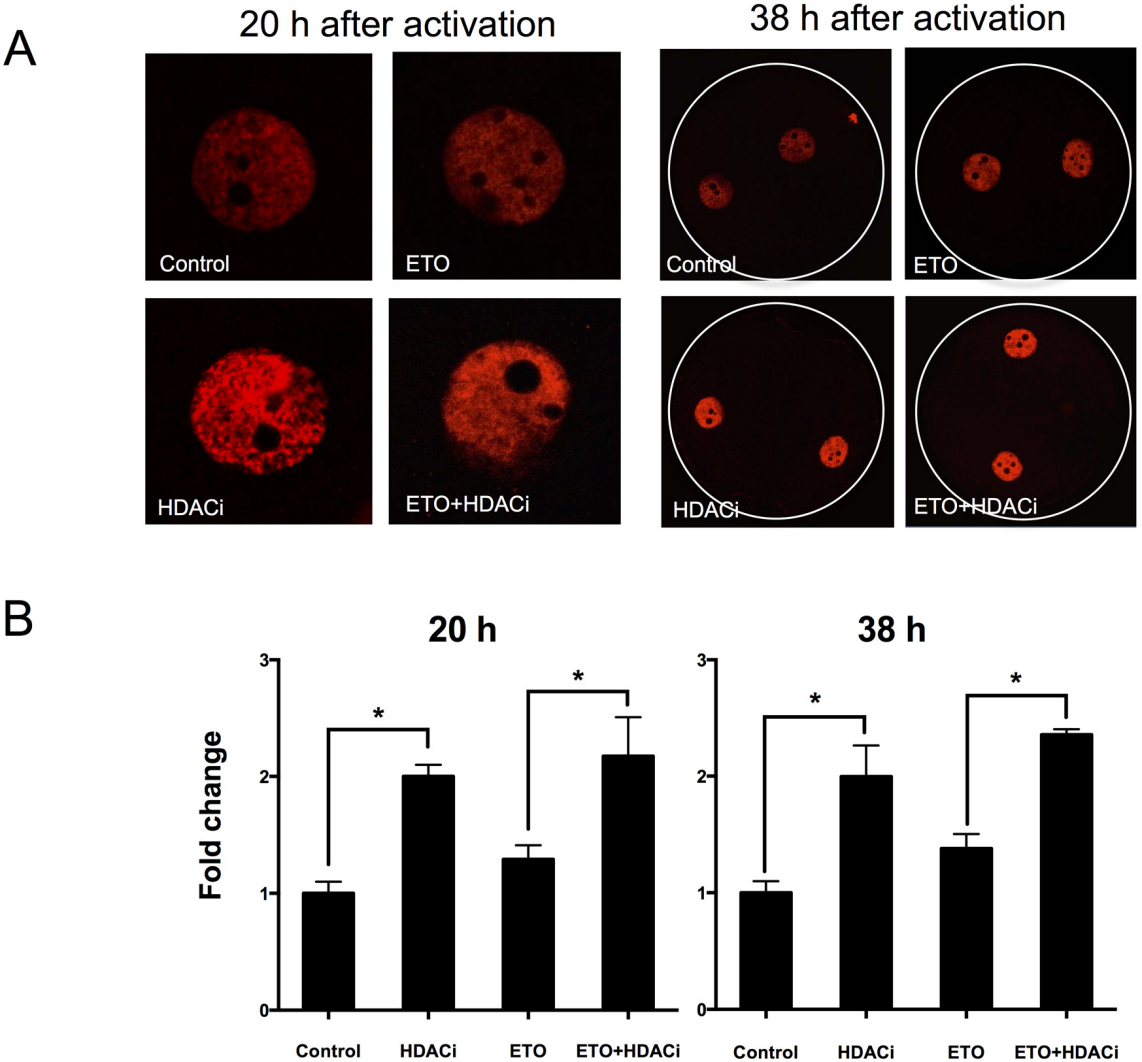
HDACi treatment increased the levels of H3K9ac at 15 h (1-cell) and 28 h (2-cell) after activation. (A) The intensity of H3K9ac at 20 h (1-cell) and 38 h (2-cell) after activation. (B) Quantification of (A) to determine the relationship between HDACi and H3K9ac. Data from (A) are presented as normalized fold change. Fold change in H3K9ac was determined using the formula F/F_con_, where F is the mean value of each group and F_con_ is the control value where no etoposide and HDACi are used. Error bars indicate SD. ETO, etoposide; * P < 0.05.

To determine the level of histone acetylation in porcine embryos treated with HDACi for 20 h, the levels of the epigenetic marker H3K9ac were measured at the one- and two-cell stages of HDACi-treated and non-treated embryos. Under DNA-damaging conditions induced by etoposide-treatment (25 μg/mL), the fold change of the fluorescence signal for H3K9ac increased at both the one-cell (2.17 *vs*. 1.29) and two-cell (2.35 *vs*. 1.38) stages in embryos treated with HDACi, compared with non-treated embryos (Fig 7).

### HDACi Treatment Positively Affects the Expression of Genes Involved in DNA Repair, Apoptosis, and Pluripotency

**Fig 8:**
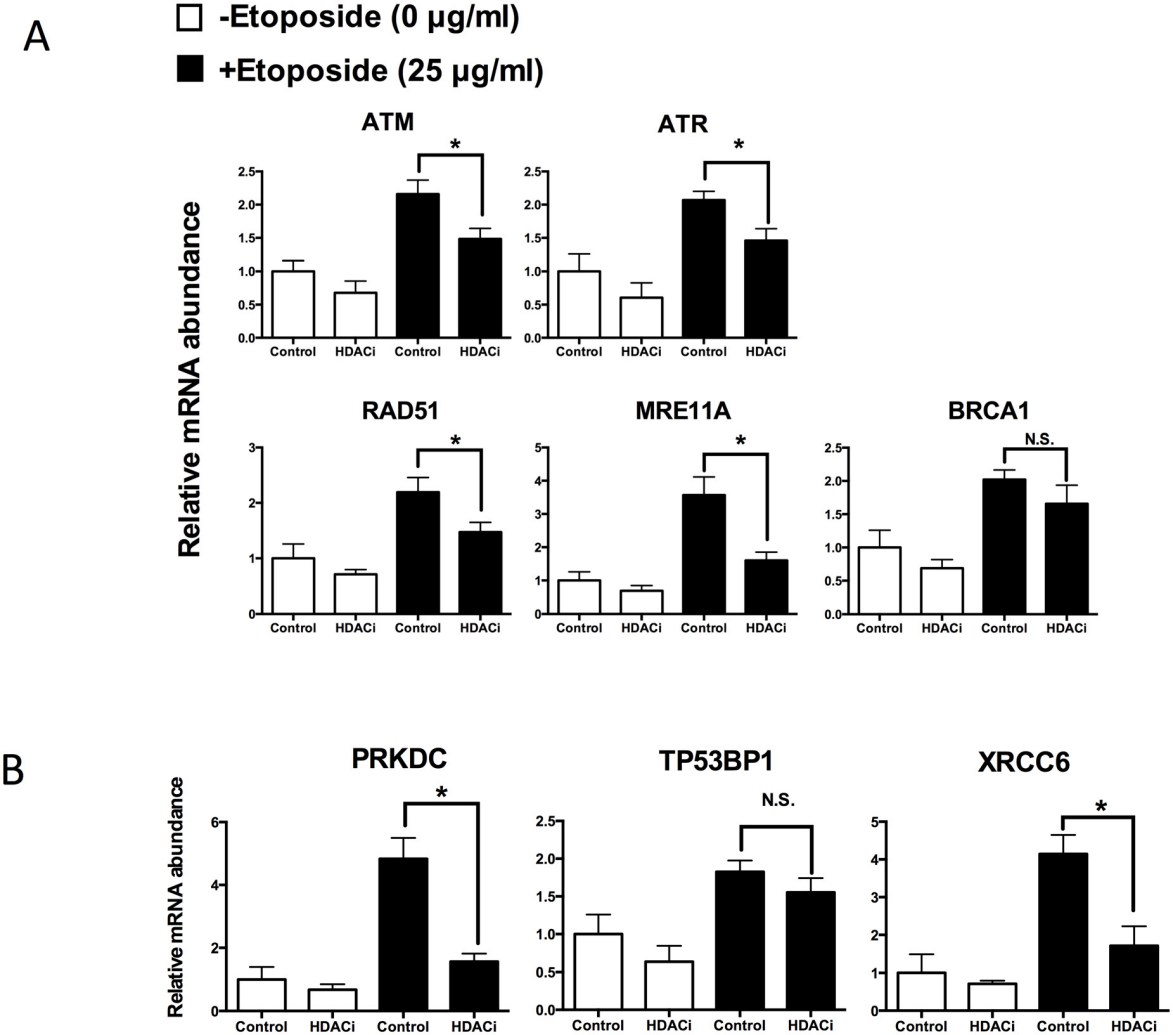
Relative mRNA abundance for genes involved in homologous recombination (A) and nonhomologous end-joining (B) pathways in day 5 embryos. Non-treated (-Etopodide; white bars) or etoposide-treated (+Etopodide; black bars) embryos were subjected to HDACi or vehicle (control). The mRNA abundance was calculated relative to the reference gene GAPDH. Data are from 3 independent replicates with 20 embryos in each group. ETO, etoposide; * P < 0.05; N.S., not statistically significant (P > 0.05).

To further explore the effect of HDACi treatment on DSB repair, the mRNA levels of DNA damage-response genes participating in the HR and NHEJ repair pathways were evaluated in 5-day-old embryos. Etoposide-treated (25 μg/mL) embryos had higher mRNA levels of genes involved in HR (*ATM*, *ATR*, *RAD51*, *MRE11A*; Fig 8A) and NHEJ (*PRKDC*, *XRCC*; Fig 8B) than non-treated embryos (control, 0 μg/mL etoposide) on Day 5. Interestingly, HDACi treatment reduced the overall expression of all assessed genes in etoposide-treated (25 μg/mL) embryos to levels that were almost equivalent to those observed in the control embryos (Fig 8). The lower expression of DNA-repair genes in the 5-day-old embryos treated with HDACi indicates that they had fewer DSBs. In contrast to findings in day-5 embryos, there was no difference in the levels of mRNA expression for any of the studied genes involved in the HR and NHEJ repair pathways in 7-day-old embryos (S1 Fig). These results suggest that embryos that developed to the blastocyst stage had completed DNA DSBs repair by Day 7.

**Fig 9:**
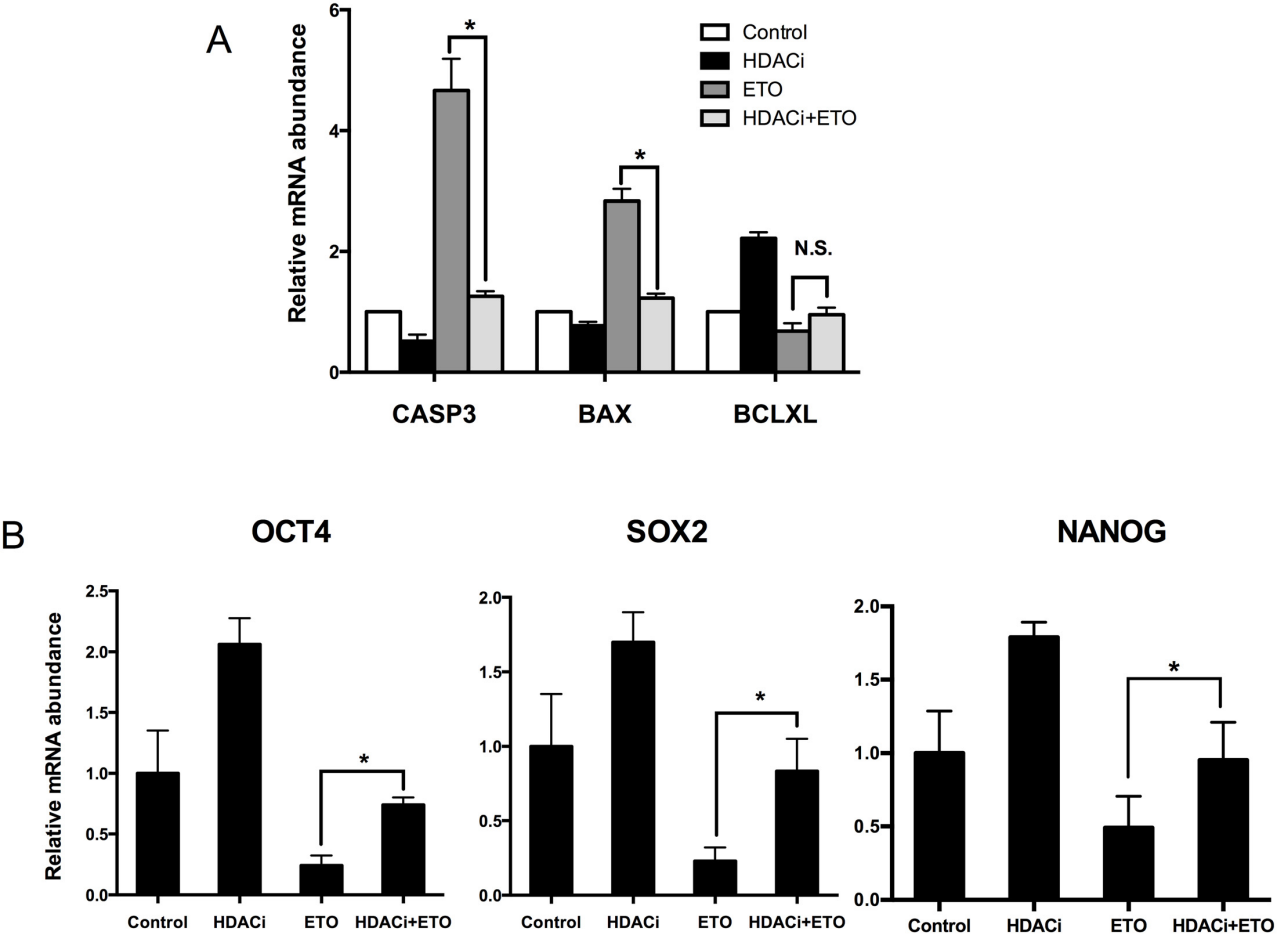
The relative expression patterns of apoptosis (A) and pluripotency (B) related genes in 7-day-old embryos. The concentration of etoposide used in this experiment was 25 μg/mL. The mRNA abundance was calculated relative to the reference gene GAPDH. Data are from 3 independent replicates with 20 embryos in each group. ETO, etoposide; * P < 0.05; N.S., not statistically significant (P > 0.05).

To investigate the effects of HDACi treatment on gene expression patterns during early embryonic development, the expression levels of three genes related to pluripotency (*OCT4*, *SOX2* and *NANOG*) and of three genes related to apoptosis (*BCLXL*, *CASP3*, and *BAX*) were determined in 7-day-old embryos. The mRNA expression levels of *CASP3* and *BAX* were significantly decreased in HDACi-treated embryos under DNA-damage conditions (25 μg/mL etoposide treatment) (Fig 9A). On the other hand, the mRNA expression levels of *OCT4*, *SOX2 and NANOG* were significantly increased in HDACi-treated embryos under the same conditions (Fig 9B).

## Discussion

In this study, we investigated the effect of etoposide-induced DNA DSBs on cell division and embryonic development in porcine early preimplantation embryos. The results indicate that etoposide-induced DNA DSBs in early embryos elicit DNA damage response, cause cell cycle delay, and lead to cell apoptosis in 5-day-old embryos. Here, we showed that caffeine (an ATM and ATR inhibitor) and KU55933 (an ATM inhibitor) effectively relieve the cell cycle blocks induced by etoposide-treatment *in vitro*. Furthermore, we found that treatment with HDACi promoted DNA damage repair and improved the development of porcine early embryos.

The DNA damage checkpoint seems to function insufficiently in porcine one- and two-cell embryos. Parthenogenesis embryos that had been treated with etoposide divided after a temporal delay of the cell division and arrested at approximately the four- or eight-cell stage. It is likely that the DSBs had not been completely repaired during cell division arrest because the embryos ceased to develop before reaching the blastocyst stage. Therefore, their cleavage prior to the completion of DNA repair suggests that the DNA damage checkpoint mechanisms do not function sufficiently. γH2AX is required to maintain repair factors at the site of DNA DSBs [29]. Of the two DNA repair mechanisms, NHEJ is active throughout the cell cycle, while HR is restricted to the S and G2 phases of the cell cycle due to the requirement for the sister chromatid as a template [30]. γH2AX seems to play an important role in both HR and NHEJ mechanisms [31]. We evaluated the function of the DNA repair mechanisms by examining the phosphorylation of H2AX after etoposide treatment. The detection of phosphorylated H2AX in etoposide-treated embryos suggests that HR and NHEJ were activated. Based on these findings, we propose that the division of etoposide-treated embryonic cells is more likely due to a lack of efficient DNA damage checkpoint mechanisms, rather than a decreased capacity for DNA repair.

The insufficient function of the DNA damage checkpoint in porcine one- and two-cell embryos may be caused by a low expression of the involved genes prior to zygotic gene activation (ZGA) [32]. Pig ZGA was confirmed to occur at the four-cell stage via genome-wide gene expression analysis, compensating for the loss of maternal transcripts [33]. Thus, if a DNA damage checkpoint gene is insufficiently transcribed, its transcripts would decrease to a low level at the one- or two-cell stage. These levels may increase again at the four-cell stage, allowing a functional DNA damage checkpoint to occur. This hypothesis is supported by the observation that etoposide-treated embryos arrest cell division at approximately the 4–8-cell stage.

A major consequence of unrepaired DNA DSBs is cell apoptosis or death. Laser microbeam-induced DNA DSBs cause embryonic cell cycle arrest and finally cell apoptosis at the blastocyst stage in mouse embryos [8]. We used TUNEL staining as a means of detecting apoptosis in the etoposide-treated embryos. In the present study, the earliest positive TUNEL signals were detected in the etoposide-treated embryos on Day 5 of *in vitro* culture, while no apoptosis was observed on Days 3 and 4. This result is similar to the results of apoptosis assays previously performed in mouse and pig somatic cell nuclear transfer (SCNT) embryos [8,34]. It is not clear why no apoptosis is observed during the earliest stages. Although every cell has an apoptotic pathway, it is known that chromatin structures change during early development and that the major onset of gene expression begins only at the four-cell embryonic stage [33]. A previous study has shown that apoptosis depends on DNA methylation and histone acetylation in early embryos [34]. Furthermore, the propensity to apoptosis is continuously counterbalanced in the cell by genes that stimulate cell survival and proliferation [34,35]. These observations confirm that embryos exhibit dramatic changes in the expression pattern of several apoptosis-related genes when apoptosis is activated during the development of early implantation embryos.

Caffeine is a well-established inhibitor of the activity of both ATR and ATM kinases, whereas KU55933 is the first potent and selective ATM inhibitor [18,20,36]. Interestingly, our results demonstrate that both caffeine (5 mM) and KU55933 (10 μM) were equally effective at reversing the cell-cycle block that was induced by etoposide treatment. These results hint that ATM plays a major role in mediating the cell-cycle block induced by etoposide treatment. A potential effector pathway is the ATM phosphorylation of CHK2 [37], which has a number of downstream target proteins capable of inducing a G1-S phase checkpoint. For instance, ATM and CHK2 cause inhibition of cell division cycle 25 homolog A (Cdc25A), which prevents the activation of Cdk2 and results in checkpoint activation [11]. However, the low levels of ATM activation in one- and two-cell embryos also indicate that the DNA damage checkpoints function insufficiently in these embryos. A possible reason for this limited ATM activity could be low ATM expression levels. This explanation was supported by a study that monitored the total levels of ATM in oocytes and blastocysts, the results of which indicated that the levels of ATM in fully grown oocytes were lower than those in growing oocytes and blastocysts [4]. These findings support the hypothesis that the lack of an efficient DNA damage checkpoint during early embryonic development is due to the ineffective activation of ATM kinase, the master regulator of the DNA damage response pathway. In addition, ATM activity is known to be influenced by chromatin structure [38], and early preimplantation embryos have a distinct chromatin configuration and histone modification [39], both of which may limit the response to DNA damage.

Caffeine and KU55933 can reverse the cell-cycle block, but they do not seem to aid in DNA repair, as evidenced by the increased apoptosis in caffeine/KU55933- and etoposide-treated embryos. We next sought to determine if HDACi treatment affects the DNA damage repair in etoposide-treated embryos. Chromatin remodeling and epigenetic changes, including histone modifications, are important parameters for cell reprogramming and normal development of SCNT embryos [40]. It is known from studies in somatic cells that histone H4 acetylation at lysine 16 is critical for DNA damage response and DSBs repair [24]. It has also been shown that H3K9Ac and H3K56Ac are reduced in response to DNA damage in human cells [27]. The HAT Gcn5 can interact with γH2AX at the sites of DSBs and then acetylate various lysine residues on histone H3 (including H3K9, H3K14, H3K18, and H3K23) [26]. More recently, a study showed that HDACi treatment after nuclear transfer enhances DSB repair and development of SCNT embryos [41]. Thus, we hypothesized that increasing histone acetylation by HDACi treatment would facilitate DNA damage repair in etoposide-treated embryos. Indeed, we have found that HDACi treatment greatly increased cell division and embryonic development to the blastocyst stage in embryos treated with etoposide. Moreover, HDACi treatment consistently reduced the γH2AX in cleaved etoposide-treated embryos. These findings indicate that HDACi treatment can facilitate DNA damage repair in etoposide-treated embryos. Although HDACi may affect other DNA functions, including transcription and replication [42], its positive effect on DSB repair seems to be an important route by which HDACi treatment improves the development of etoposide-treated embryos.

To further evaluate the effect of HDACi treatment on DSB repair, we assessed the expression of several genes involved in either the HR (*ATM*, *ATR*, *RAD51*, *MRE11A*, *BRCA1*) or the NHEJ (*53BP1*, *PRKDC*, *XRCC6*) pathways in etoposide-treated embryos at Day 5 of development. Transcript levels of the genes involved in the two DNA damage repair pathways were increased in these embryos, confirming that both pathways are activated in etoposide-treated embryos. In this study, we observed an overall tendency for a decreasing expression of these genes when the embryos were exposed to HDACi. The lower expression of DNA-repair genes indicates that Day 5 etoposide-treated embryos treated with HDACi have fewer DSBs. In contrast to Day 5 embryos, we observed that none of the assessed genes were differentially expressed in 7-day-old blastocysts, even in those etoposide-treated embryos. This finding is in line with previous observations in embryos produced by somatic cell nuclear transfer, whereas the induction of DNA damage by UV treatment significantly reduced embryo development, but those that achieved the blastocyst stage had similar number of DSBs and levels of transcripts encoding DNA repair genes [41]. These findings support the hypothesis that only the embryos with less DNA DSBs and/or superior capacity for DNA repair are able to progress to the blastocyst stage. The fact that HDACi could rescue the development of a higher proportion of etoposide-treated embryos, and that important genes involved in apoptosis pathways were normally expressed in the developing blastocysts, further indicates that HDACi treatment promotes DSB repair during embryo development.

In summary, the data obtained in this study indicate that etoposide-induced DNA DSBs can alter the kinetics of embryo cell cleavage and development to the blastocyst stage. However, DNA damage checkpoint function seems insufficient in one- and two-cell embryos, and this appears to be mainly due to limited ATM activation. The earliest TUNEL-positive cells were detected in embryos after 5 days of *in vitro* culture, which indicates that DNA DSBs cause embryonic cell cycle delay and finally cell apoptosis at a particular time-point. Caffeine and KU55933 were equally effective in reversing the etoposide-induced cell-cycle block and allowed porcine early embryos to progress through several further cell cycles. Moreover, we have shown that HDACi exposure can rescue the development of embryos with DSBs by improving DNA damage repair during early embryo development. In conclusion, the findings of this study revealed a negative correlation between the occurrence of DSBs and embryo cleavage kinetics, and the capacity of the embryo to develop to the blastocyst stage. Chromatin remodeling is important for the promotion of DNA damage repair and the preservation of genome integrity for normal embryo development.

## Supporting Information

S1 Fig Relative mRNA abundance for genes involved in homologous recombination (A) and nonhomologous end-joining (B) pathways in day 7 embryos. Non-treated (-Etopodide; white bars) or etoposide-treated (+Etopodide; black bars) embryos were subjected to HDACi or vehicle (control). The mRNA abundance was calculated relative to the reference gene GAPDH. Data are from 3 independent replicates with 20 embryos in each group. ETO, etoposide.
(TIFF)
