# Supplementary figures and images for: Effect of ATM and HDAC Inhibition on Etoposide-Induced DNA Damage in Porcine Early Preimplantation Embryos
Source: PLoS One. 2015 Nov 10;10(11):e0142561. doi: 10.1371/journal.pone.0142561 (PMC4640854; doi:10.1371/journal.pone.0142561)

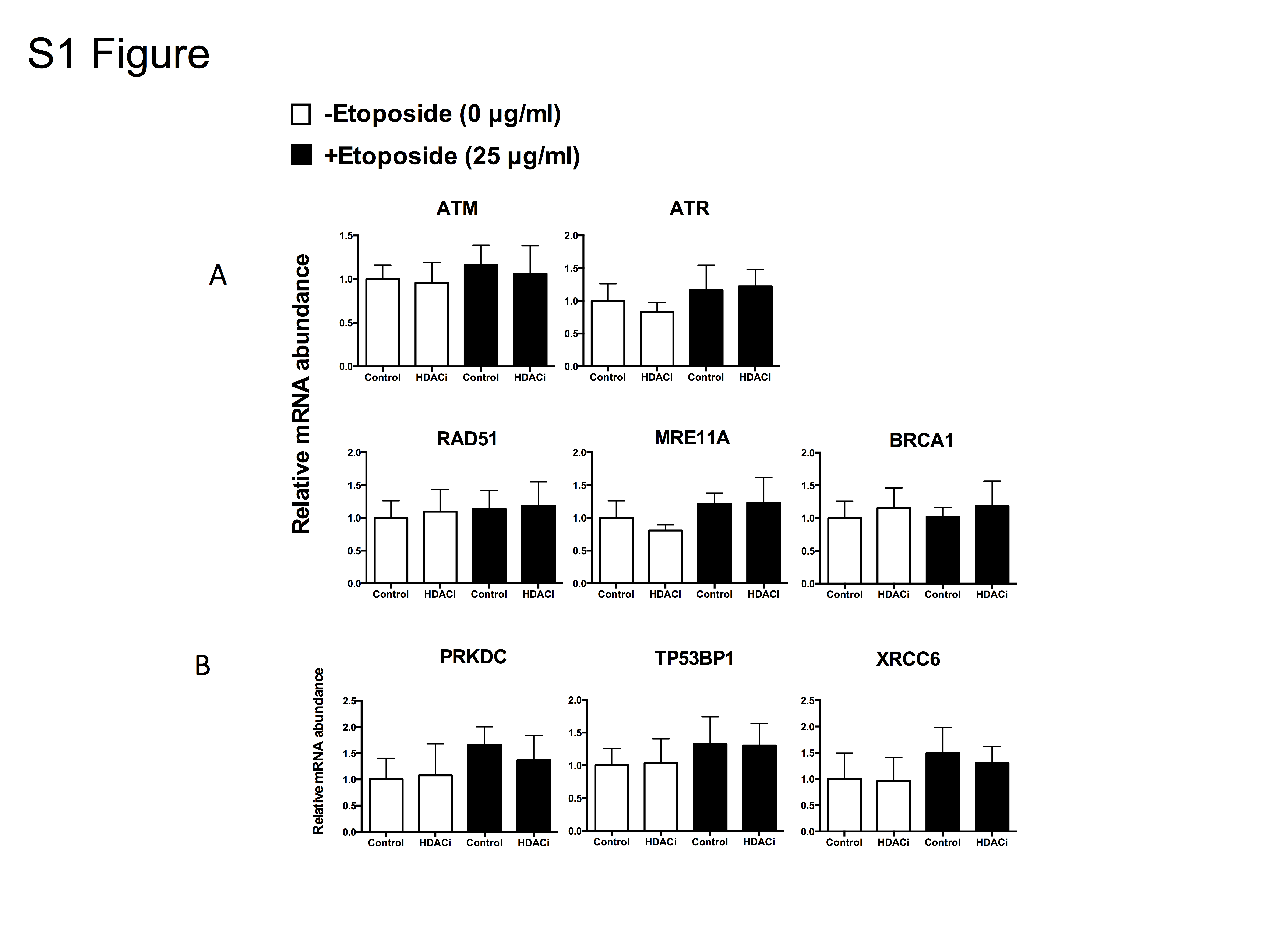

Supplement: S1 Fig — Non-treated (-Etopodide; white bars) or etoposide-treated (+Etopodide; black bars) embryos were subjected to HDACi or vehicle (control). The mRNA abundance was calculated relative to the reference gene GAPDH. Data are from 3 independent replicates with 20 embryos in each group. ETO, etoposide. (TIFF) [file pone.0142561.s001.tiff]
